# A novel desmin (*DES*) indel mutation causes severe atypical cardiomyopathy in combination with atrioventricular block and skeletal myopathy

**DOI:** 10.1002/mgg3.358

**Published:** 2017-12-23

**Authors:** Ilona Schirmer, Mareike Dieding, Bärbel Klauke, Andreas Brodehl, Anna Gaertner‐Rommel, Volker Walhorn, Jan Gummert, Uwe Schulz, Lech Paluszkiewicz, Dario Anselmetti, Hendrik Milting

**Affiliations:** ^1^ Erich and Hanna Klessmann Institute for Cardiovascular Research & Development (EHKI) Heart and Diabetes Centre NRW University Hospital of the Ruhr‐University Bochum Bad Oeynhausen Germany; ^2^ Faculty of Physics, Experimental Biophysics and Applied Nanoscience Bielefeld Institute for Nanoscience (BINAS) Bielefeld University Bielefeld Germany

**Keywords:** cardiomyopathy, cardiovascular genetics, desmin, intermediate filament proteins, skeletal myopathy

## Abstract

**Background:**

*DES* mutations cause different cardiac and skeletal myopathies. Most of them are missense mutations.

**Methods:**

Using a next‐generation sequencing cardiac 174 gene panel, we identified a novel heterozygous in‐frame indel mutation (*DES*‐c.493_520del28insGCGT, p.Q165_A174delinsAS) in a Caucasian patient with cardiomyopathy in combination with atrioventricular block and skeletal myopathy. This indel mutation is located in the coding region of the first exon. Family anamnesis revealed a history of sudden cardiac death. We performed cell transfection experiments and in vitro assembly experiments to prove the pathogenicity of this novel *DES* indel mutation.

**Results:**

These experiments revealed a severe filament formation defect of mutant desmin supporting the pathogenicity. In addition, we labeled a skeletal muscle biopsy from the mutation carrier revealing cytoplasmic desmin positive protein aggregates. In summary, we identified and functionally characterized a pathogenic *DES* indel mutation causing cardiac and skeletal myopathy.

**Conclusion:**

Our study has relevance for the clinical and genetic interpretation of further *DES* indel mutations causing cardiac or skeletal myopathies and might be helpful for risk stratification.

## INTRODUCTION

Desmin, encoded by *DES*, is the major component of muscle intermediate filaments (IF) and is important for the structural integrity of muscle cells. IFs connect different cell organelles like desmosomes, costameres, and Z‐disks with the cytoskeleton (Goldfarb & Dalakas, 2009). Desmin consists of a central rod domain flanked by non‐α‐helical head and tail domains (Köster, Weitz, Goldman, Aebi, & Herrmann, 2015). *DES* mutations cause a broad range of skeletal myopathies (Li & Dalakas, 2001) and different cardiomyopathies (Brodehl, Dieding, et al., 2016; Taylor et al., 2007). The majority of *DES* mutations are missense mutations within the central rod domain causing cytoplasmic desmin aggregates (Brodehl et al., 2012), whereas nonsense, insertions, deletions (Muñoz‐Mármol et al., 1998) or combined insertions and deletions (indel) mutations (Cao et al., 2013) are rare.

**Figure 1:**
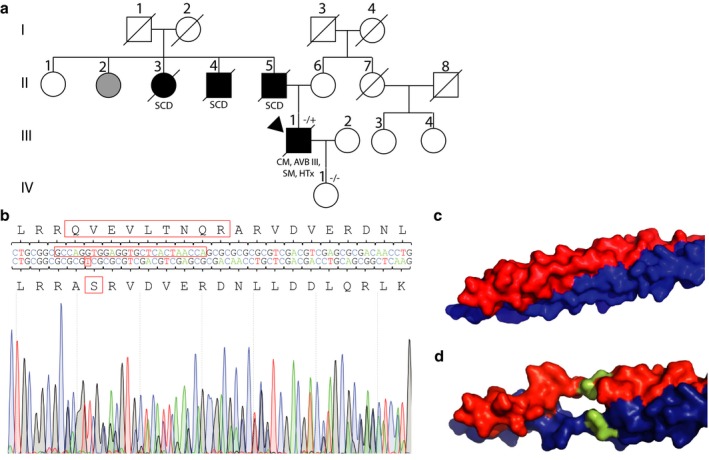
(a) Pedigree of the family. Circles represent females, squares males, slash denotes deceased. Black filled symbols indicate a clinical phenotype of cardiomyopathy. Gray filled symbols indicate individuals with a suspected clinical phenotype. CM cardiomyopathy; SCD sudden cardiac death; AVB atrioventricular block; SM skeletal myopathy; HTx heart transplantation; −/+ heterozygous allele; −/− wild type for both *DES* alleles. The index patient is marked with an arrow. (b) Electropherogram of *DES*‐c.493_520del28insGCGT (reference NM_001927.3). Molecular structure of wild‐type (c) and mutant (d) desmin dimer modeled using Swiss‐Model (PDB ID: 3uf1; Kuzin et al., 2011)

The index patient (Figure 1a, III‐1) received his first cardiac diagnosis of suspected myocarditis aged 16 years. At the age of 41 years atrioventricular block was diagnosed and a pacemaker was implanted. Two years later an implantable cardioverter defibrillator was implanted as a secondary prevention because of sustained ventricular tachycardia. The systolic function of the left ventricle decreased systematically over time achieving finally 20%. Interestingly, the left ventricular diameter remained normal. The clinical status of the patient deteriorated constantly and finally he received heart transplantation aged 57 years. Several members of the paternal family branch died by sudden cardiac death (Figure 1a). The patient's father (II‐5), his uncle (II‐4) and one aunt (II‐3) died at the age of 44, 53, and 63 years. Presumably, the second aunt (II‐2) was also affected. Family anamnesis supports an inherited genetic etiology.

We obtained written consent of the probands to this study and genotyped the index patient using the TrueSight Cardio Sequencing Kit (Illumina, San Diego, USA; 174 genes). The identified variants including *DES*‐p.Q165_A174delinsAS were listed in Table S1. The genetic etiology of cardiomyopathies is broad and more than 40 different genes are involved in cardiomyopathies. Based on the minor allele frequency, pathogenicity of all other nonsynonymous variants was excluded. Sanger sequencing revealed the *DES*‐c.493_520del28insGCGT genotype for the index patient (Figure 1b). Compound heterozygosity could be excluded by cloning and sequencing of this exon. *DES*‐c.493_520del28insGCGT was submitted to ClinVar (https://www.ncbi.nlm.nih.gov/clinvar/variation/265817/). The patient's healthy daughter (IV‐1) did not carry this mutation (Figure 1a). Genomic DNA from further family members was not available limiting a cosegregation analysis. Public genetic population databases (ExAc Browser, http://exac.broadinstitute.org; Exome Variant Server, http://evs.gs.washington.edu/EVS; Human Mutation Gene Database, http://www.hgmd.cf.ac.uk; October 2017) do not contain this mutation. Absence of a genetic variant in population databases is, according to guidelines of the American College of Medical Genetics and Genomics (ACMG), a moderate criterion for pathogenicity (Richards et al., 2015).

A meta‐analysis of *DES* mutations revealed that about 50% of the *DES* mutation carriers developed cardiomyopathy and about 60% had a conduction disease with atrioventricular block (van Spaendonck‐Zwarts et al., 2011). Desmin is highly expressed in cells of the Purkinje system (Schrickel et al., 2010), which may explain the high association of *DES* mutations with conduction diseases. However, the majority of *DES* mutations are missense mutations (Azzimato, Gennebäck, Tabish, Buyandelger, & Knöll, 2016) and no further *DES* indel variants were described before. Because the reported indel mutation is in‐frame, functional consequences are not obvious. Therefore, we decided to investigate the molecular defects by homology‐modeling using Swiss‐Model (https://swissmodel.expasy.org/; Figure 1c,d) and functional in vitro analysis. Molecular modeling using a structure of the homologous protein vimentin as a template (3uf1; http://www.rcsb.org; Kuzin et al., 2011) revealed a significant gap in the mutant desmin dimer leading to a twist of the α‐helix. In consequence of *DES*‐c.493_520del28insGCGT, eight amino acids are deleted in desmin. This causes a shift of the coiled coil heptad sequence. Consequently the two α‐helices are displaced destroying the hydrophobic seam. This is in good agreement with the in silico prediction using Provean (Score: −36.34; deleterious; −2.5 cut off; http://provean.jcvi.org/index.php) (Choi, Sims, Murphy, Miller, & Chan, 2012). The in silico prediction is a supportive criterion for pathogenicity (Richards et al., 2015).

**Figure 2:**
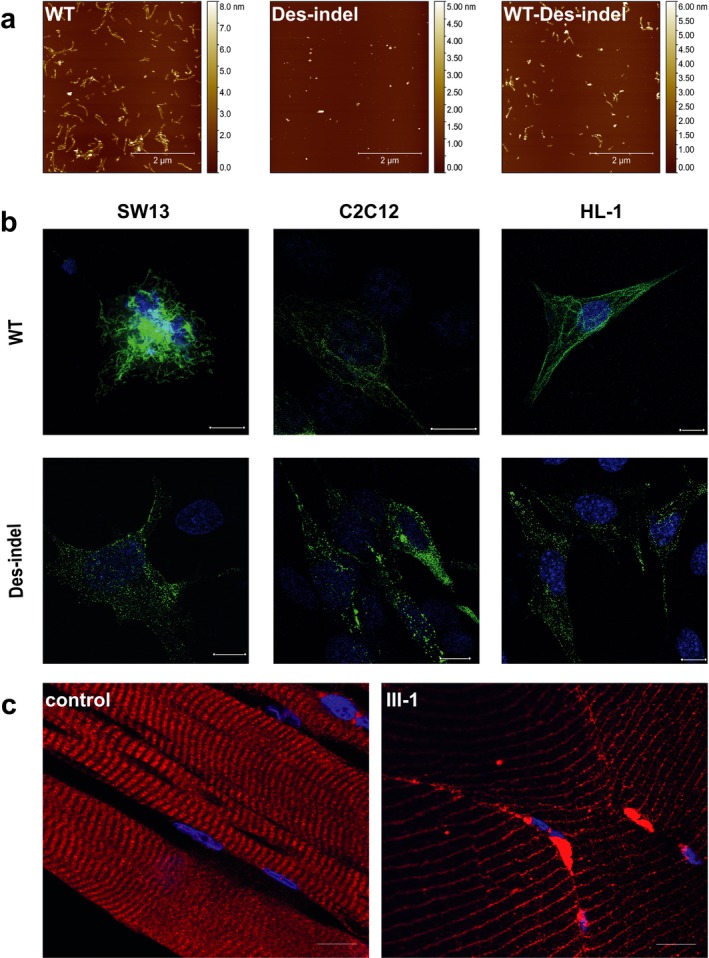
(a) Desmin filament assembly of recombinant wild‐type and mutant desmin was investigated using atomic force microscopy. Representative topographic images of desmin‐wild‐type, desmin‐p.Q165_A174delinsAS and a coassembled equimolar mixture of both are shown. Recombinant desmin molecules were purified by ionic exchange and immobilized metal affinity chromatography. The assembly was initiated by addition of sodium chloride as recently described (Kreplak & Bär, 2009). Of note, in comparison to the control experiment using wild‐type desmin, the mutant desmin‐p.Q165_A174delinsAS does not form filaments and inhibited even the coassembly of both forms indicating an intrinsic protein filament assembly defect. (b) Cell transfection studies. Wild‐type desmin (green) formed regular intermediate filaments, whereas desmin‐p.Q165_A174delinsAS formed mainly cytoplasmic aggregates of different shape and size. Scale bars represent 10 μm. (c) Desmin localization in a skeletal muscle biopsy of a control person and the index patient III‐1. Nuclei were stained with DAPI. Scale bars represent 10 μm

To prove this hypothesis, we analyzed the functional impact of *DES*‐c.493_520del28insGCGT using recombinant mutant desmin and also by cell transfection experiments. Therefore, we expressed mutant and wild‐type desmin in *escherichia coli* (BL21‐Star) and purified recombinant desmin by ionic exchange and immobilized metal affinity chromatography. Purified recombinant desmin was investigated on its ability to form filaments at the molecular level by atomic force microscopy (AFM) as previously described (Brodehl et al., 2012). These experiments revealed a severe intrinsic filament assembly defect for the mutant protein. Only small protein aggregates were detected in case of mutant desmin, whereas, wild‐type desmin forms regular IFs (Figure 2a). Our experiments indicate that the oligomerization steps of mutant desmin into unit length filaments and mature IFs are affected by the mutation. Because the patient carried a heterozygous *DES* mutation, we used in addition a mixture of mutant and wild‐type desmin (Figure 2a). Filaments as well as protein aggregates were detected in the case of the equimolar mixture (Figure 2a).

To verify the filament formation defect, we transfected HL‐1, C2C12 and SW13 cells with mutant and wild‐type desmin expression constructs. HL‐1 cells were used as a model for cardiomyocytes (Claycomb et al., 1998) and the C2C12 line was used as a model for skeletal myocytes as previously described (McMahon et al., 1994). C2C12 and SW13 cells were cultured under standard conditions (5% CO_2_, 37°C, humidified incubator) in DMEM supplemented with 10 % fetal bovine serum and penicillin/streptomycin. HL‐1 cells were cultured as previously described (Claycomb et al., 1998). SW13 cells do not express any endogenous cytoplasmic IF proteins and are therefore a suited cell culture model to investigate IFs (Sarria, Lieber, Nordeen, & Evans, 1994). The cell culture transfection experiments revealed that mutant desmin forms mainly cytoplasmic aggregates, whereas wild‐type desmin forms regular IFs (Figure 2b).

Therefore, our functional in vitro analysis supports the pathogenicity of *DES*‐c.493_520del28insGCGT. Beside mutations in *DES*, mutations in several other genes like *CRYAB* (Brodehl et al., 2017), *MYOT* (Maerkens et al., 2016), *FLNC* (Brodehl, Ferrier, et al., 2016) or *BAG3* (Selcen et al., 2009) are associated with pathogenic protein aggregation in cardiomyocytes. Interestingly, there is an overlap of cardiac and neurologic diseases. For example, Alexander disease (MIM, 203450) is caused by mutations in *GFAP,* encoding glial fibrillary acidic protein (Brenner et al., 2001). GFAP is homologous to desmin. It is mainly expressed in different cell types of the central nervous system and *GFAP* mutations cause also cytoplasmic protein aggregation (Quinlan, Brenner, Goldman, & Messing, 2007).

Furthermore, we analyzed a skeletal muscle biopsy of the mutation carrier (III‐1) by immunohistochemistry. In the control tissue desmin was localized as expected at the Z‐bands, whereas desmin formed partially cytoplasmic aggregates in myocytes of the mutation carrier (Figure 2c). These experiments are in good agreement with the cell transfection experiments and demonstrate that mutant desmin accumulates also in vivo supporting the pathogenicity of *DES*‐c.493_520del28insGCGT (strong criterion; ACMG guidelines; Richards et al., 2015).

In summary, our report describes a novel *DES* indel mutation associated with an atypical cardiomyopathy, characterized by systolic dysfunction without ventricular dilation, in combination with atrioventricular block, ventricular arrhythmia and skeletal myopathy. *DES*‐c.493_520del28insGCGT fulfills one supportive, one moderate and one strong criterion for pathogenicity but no criterion for its classification as benign. In conclusion, *DES*‐c.493_520del28insGCGT has to be classified according to the ACMG guidelines as a likely pathogenic mutation. Our report may help for risk stratification of further *DES* indel mutations.

## CONFLICT OF INTEREST

None.

## Supporting information


** **
