# Supplementary material for: A novel desmin (DES) indel mutation causes severe atypical cardiomyopathy in combination with atrioventricular block and skeletal myopathy
Source: Mol Genet Genomic Med. 2017 Dec 23;6(2):288–93. doi: 10.1002/mgg3.358 (PMC5902401; doi:10.1002/mgg3.358)
Supplement: Supplementary file 1 [file MGG3-6-288-s001.docx]

**Table S1. Overview about non-synonymous sequence variations identified in the index patient (III:1)**

| **Chromosome** | **Position** | **Reference** | **Alteration** | **MAF** | **Gene** |
| --- | --- | --- | --- | --- | --- |
| 1 | 116243874 | ATCG | A | 0.005493 | *CASQ2* |
| 2 | 73613031 | T | TGGA | 0.03701 | *ALMS1* |
| 2 | 73675227 | T | TCTC | 0.6384 | *ALMS1* |
| 2 | 21235475 | T | C | 0.9999 | *APOB* |
| 2 | 189875421 | T | G | 0.9985 | *COL3A1* |
| 2 | 220283674 | CGCCAGGTGGAGGTGCTCACTAACCA | CGCCAGGTGGAGGTGCTCACTAACCA | Novel | *DES* |
| 2 | 220283704 | G | GT | Novel | *DES* |
| 2 | 179621477 | C | T | 0.9997 | *TTN* |
| 2 | 179444768 | C | G | 0.9986 | *TTN-AS1,TTN* |
| 3 | 123451773 | G | C | 0.9996 | *MYLK* |
| 7 | 91714911 | C | T | 0.9962 | *AKAP9* |
| 10 | 112572458 | G | C | 0.9968 | *RBM20* |
| 11 | 111782297 | G | A | 0.000447 | *CRYAB* |
| 11 | 6630028 | TC | T | 1.0 | *ILK* |
| 12 | 2791205 | A | G | 0.9999 | *CACNA1C* |
| 15 | 48807637 | C | T | 1.000 | *FBN1* |
| 18 | 29101156 | T | G | 0.005377 | *DSG2* |

MAF = minor allele frequency.
